# A common risk factor strategy for combating childhood oral diseases and malnutrition in Kalpetta, India

**DOI:** 10.3389/froh.2026.1673066

**Published:** 2026-02-16

**Authors:** Chandrashekar Janakiram, Venkitachalam Ramanarayanan, Anju James, Arya S. Raj, Anna Maria Babu, Sanjeev Vasudevan

**Affiliations:** Amrita School of Dentistry, Amrita Vishwa Vidyapeetham, Kochi, India

**Keywords:** childhood dental caries, common risk factor approach, dental caries, malnutrition, oral health

## Abstract

**Aim/purpose:**

Childhood dental caries and malnutrition share a bi-directional relationship and represent significant public health challenges. This study aimed to evaluate the effectiveness of a Common Risk Factor Strategy-based intervention in improving childhood oral health and nutrition among preschool and primary school tribal children in Kerala, India.

**Methods:**

This pre-post quasi-experimental study was conducted among tribal children and their caregivers. Baseline assessments included knowledge of oral health, childhood dental caries, and nutrition, along with evaluations of oral health and nutritional status using validated questionnaires and indices. The intervention comprised oral hygiene awareness, dietary counseling, caries prevention through fluoride varnish application, arrest of cavitated lesions using silver diamine fluoride (SDF), curative dental treatment, and the regular provision of toothpaste and toothbrushes. The effectiveness of these interventions was assessed after one year.

**Results:**

A total of 95 children (mean age: 4.47 ± 1.42 years) and their caregivers participated in the study. Post-intervention, there was a significant increase in mean KAP childhood caries assessed using 24 point scale [mean difference (MD): 14.52 ± 5.01] and nutrition knowledge assessed using 10 point scale (MD: 3.67 ± 3.42). The mean number of decayed teeth significantly decreased from 7.71 ± 3.59 to 1.43 ± 0.53, accompanied by notable improvements in oral hygiene and nutritional indicators. Among children under 5 years, weight-for-age and weight-for-length Z-scores improved significantly following the intervention (*p* < 0.05).

**Conclusions:**

This study highlights the effectiveness of an integrated medical-dental approach in addressing childhood oral health and malnutrition. Strengthening early prevention strategies through the Common Risk Factor Approach can serve as a sustainable model to improve overall child health and well-being in vulnerable populations. Prioritizing such holistic interventions in public health policies can be a game-changer in reducing health inequities.

## Introduction

Despite sustained initiatives over the past five decades to control dental caries, the condition continues to affect a substantial share of the global population (1, 2). The World Health Organization has recognized dental caries as a pressing public health concern, given its wide-ranging social, economic, and environmental consequences (3).Childhood dental caries is a preventable condition that continues to affect millions of children, particularly in low-resource settings (4). Its severe forms can result in significant pain, loss of primary teeth, and disruption in eating and sleeping patterns, ultimately compromising a child's growth, development, and psychosocial well-being (5). Secondary findings reveal that untreated oral diseases have a higher economic impact than preventive measures (6). When untreated, childhood caries can contribute to insufficient nutrient intake due to oral discomfort, which may eventually lead to malnutrition. A systematic review, highlighted a link between dental caries and growth stunting, suggesting that reduced salivary function and enamel formation defects in stunted children may increase their susceptibility to caries (7).

Malnutrition, specifically undernutrition shares multiple etiological factors with dental caries, such as poor dietary practices, inadequate access to health services, and broader socioeconomic inequalities (8). The chronic nature of malnutrition during childhood, extending into adolescence, impairs physiological processes such as salivary flow, thereby increasing the susceptibility to caries (9). Nutritional deficiencies, particularly in calcium, iron, vitamin D, and albumin, also contribute to enamel hypoplasia, which predisposes children to post-eruptive caries (10). The interaction between dental caries and malnutrition is thus complex and bi-directional, often exacerbated by coexisting health conditions and environmental determinants (11).

Traditional oral health education approaches often operate in silos, disconnected from broader health promotion strategies, and typically emphasize individual behavior change. Such models are frequently criticized for their inefficiency, redundancy, and failure to address the broader social determinants of health (12). In contrast, the Common Risk Factor Approach (CRFA) offers a holistic alternative by targeting shared determinants of multiple health conditions, including childhood dental caries and malnutrition through integrated and context-sensitive interventions (13). CRFA is particularly relevant in the context of India's tribal populations where multiple health conditions often coexist and are exacerbated by structural and environmental vulnerabilities.

The CRFA takes into account the interconnectedness of risk factors such as tobacco use, poor diet, stress, and inadequate hygiene each of which contributes not only to oral diseases but also to noncommunicable diseases (NCDs) like cardiovascular diseases, diabetes, and obesity. Recent literature supports the molecular and cellular basis of CRFA in the management of both NCDs and periodontal disease, highlighting its foundational role in addressing systemic and oral health concurrently (14). The CRFA has been recognized as a pragmatic, equitable approach, enabling integration of preventive strategies across medical and dental disciplines to mitigate chronic disease burden at the community level (15).

Furthermore, medical-dental integration an essential component of CRFA has emerged as a key strategy to promote equitable healthcare delivery, especially in underserved populations. In the Indian context, integrating dental care into the broader healthcare system is particularly important given the high burden of oral diseases and the limited access to dental services among rural and tribal communities (15). Medical-dental integration not only bridges systemic gaps in healthcare access but also facilitates early diagnosis and intervention for diseases that share common risk profiles.

A study by Puzhankara et al. (16) developed a knowledge, attitude, and practice (KAP) evaluation tool to assess the effectiveness of CRFA in managing NCDs and periodontal diseases among medical and dental professionals. The study underscored the critical need for interprofessional education and collaboration, which can also be extended to address dental caries and malnutrition through a unified framework. By incorporating the CRFA, the intervention is likely to enhance provider awareness, empower caregivers, and foster sustainable health behaviors in the community.

The key concept underlying the integrated CRFA is that promoting general health by controlling a small number of risk factors may have a major impact on a large number of diseases at a lower cost, greater efficiency and effectiveness than disease specific approaches (13). The scientific literature indicates that although only a few ongoing studies worldwide are currently applying the CRFA to improve health outcomes, its use within oral health research is even more limited. A prospective cohort study in Australia has adopted the CRFA to address socio-economic disparities in preschool children's oral health, aiming to provide high-level evidence on how socio-environmental factors affect oral health and its association with childhood overweight (17). A randomized controlled trial has been initiated in the USA employing a multi-level strategy based on the Common Risk Factor Approach CRFA to reduce the risk of pediatric obesity and dental caries among South Asian (SA) immigrant children. These children are considered high-risk due to a disproportionate impact of these conditions on low-income populations and the presence of shared risk behaviors, such as certain feeding practices. The study, titled CHALO (“Child Health Action to Lower Oral Health and Obesity”), aims to effectively prevent or reduce the incidence of these diseases during infancy and childhood (18).

The World Health Organization recognizes that a public health approach focused on primary prevention is the most cost-effective, affordable, and sustainable strategy to address the global epidemic of chronic diseases. A key advancement in shaping integrated health policy is the adoption of the common risk factor approach for chronic disease prevention (13). Over the past decade, the Common Risk Factor Approach (CRFA) has increasingly gained global recognition, evidenced by its extensive implementation across a range of health promotion initiatives.

In India, the tribal or indigenous population commonly referred to as *Adivasis* represents a marginalized demographic group that is disproportionately affected by health disparities. Compared to national averages, tribal populations experience higher mortality, greater prevalence of tuberculosis, anaemia, and undernutrition (19). These communities often face compounded challenges such as food insecurity, poor hygiene, substance abuse, and limited access to quality healthcare (20). Structural inequities, including the privatization of healthcare and the declining efficiency of public health systems, further exacerbate their vulnerability. About 71% of parents of tribal children had education only up to the middle school level, indicating limited educational opportunities within this population (21).

Approximately 4.7 million tribal children in India suffer from childhood dental caries and chronic undernutrition, affecting their survival, educational attainment, and long-term productivity (22). A study among 2–5-year-old tribal children reported a mean decayed, missing, and filled teeth (dmft) score of 4.13 (SD = 0.73), underscoring the early and widespread nature of dental caries in this population (23). Socioeconomic status and income levels are key determinants of childhood dental caries risk in such settings (24).

Despite its potential, the application of CRFA to childhood oral health and malnutrition remains underexplored in tribal contexts within India. Although previous research has examined CRFA in various health domains, there is a marked gap in its implementation and evaluation among indigenous communities, particularly concerning childhood dental caries. This lack of evidence hinders the development of culturally tailored interventions that address the unique challenges faced by tribal populations.

This study aims to evaluate the effectiveness of a CRFA-based intervention in improving oral health and nutritional outcomes among tribal preschool and primary school children in Kalpetta, Kerala. By investigating this integrated model in a tribal setting, the study seeks to inform the development of scalable public health strategies that can reduce health disparities and promote equity across similarly underserved communities. Additionally, it contributes to the growing body of literature supporting medical-dental integration and the CRFA in improving health outcomes, particularly in marginalized and resource-limited populations.

## Methodology

### Study design

A quasi-experimental study with a pre-post design, without a control group, was conducted to evaluate the impact of a multi-component intervention on the oral health and nutritional status of indigenous children, along with the knowledge, attitudes, and practices (KAP) of their parents/caregivers. The study was implemented at a Primary Health Centre in Kalpetta, Wayanad District, Kerala, India.

Given the ethical and practical constraints of random assignment in this vulnerable population, participants were enrolled through non-random purposive sampling based on eligibility criteria, and all received the intervention.

- Child age between 6 months to 10 years.
- Parent/caregivers able to understand the importance of healthy nutritional practices to prevent dental caries and improve their child's nutritional status.
- Willingness of caregivers to participate in follow-up assessments.
- Ability and willingness to comply with all study requirements.

Exclusion criteria were:

- Refusal to provide consent or participate in interventions.
- Presence of chronic or severe medical conditions in the child (e.g., congenital heart disease or other disorders that could confound outcomes or affect participation).

### Sample size

Assuming an effect size of 30% increase in new caries increment, with a power 80% and level of significance at 5% (two-sided), a minimum of 90 participants were required and 95 participants were included for the study.

### Study procedure

**Figure 1:**
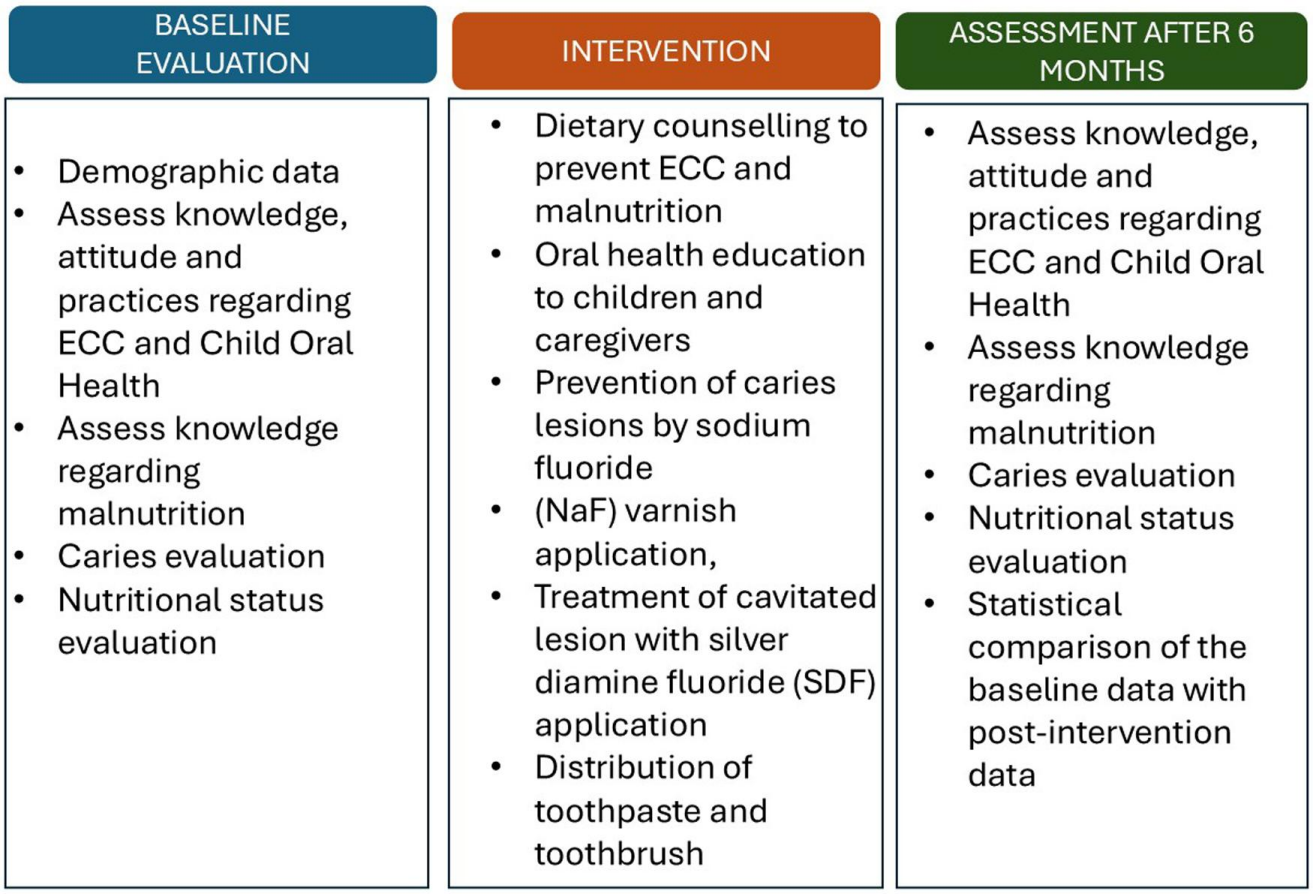
Details of the baseline evaluation, interventions, and assessment.

The study involved a) baseline assessment of the KAP of the parents/caregivers along with oral health status of the children, followed by b) appropriate intervention to improve the dental health and nutritional status of the children and finally, c) reassessment of the KAP at the end of 1 year (Figure 1).

#### KAP assessment

The KAP of the parents/caregivers were assessed using validated questionnaires designed for assessing knowledge, attitude and practice of childhood dental caries (25), child oral health (26) and malnutrition (27).

#### KAP of childhood dental caries

The knowledge of parents using 13 items which covered general knowledge about the amount of toothpaste applied according to the age, effect of mother's diet on her child's oral health, frequency of canned juices and sugary intake, the time of the first dental visit, the importance of fluoride, the appearance of white lines/spots on teeth surfaces, and preventive measures. Options for each item were “yes,” “no,” and “I don't know,” where “yes” corresponds to a positive knowledge and scored it as 1.

The second domain assessed attitudes using six items which covered parents' responsibility toward their children oral health, the effect of prolonged and frequent breast feeding, providing fresh juices frequently, dental checkup once the first tooth erupts, and parent/caregiver help for their children oral hygiene. A five-point Likert scale options were used ranging from “strongly agree” to “strongly disagree” where “strongly agree” or “agree” reflect positive attitude and is scored as one.

The third domain assessed practices using five items which covered practices related to balanced diet and its relation to their children oral health, providing breastfeeding/bottle feeding during bedtime, need of efforts to improve their knowledge in oral health, and the necessity in dental cleaning after each meal. Similar to attitudes domain, the same five-point Likert scale options were used (Supplementary File S1).

#### KAP on child oral health

This questionnaire consists of 25 questions related to knowledge, attitude and practices toward children's oral health. Out of which 10 questions were related to knowledge, six were related to attitude, and nine were related to practices, and four were general questions. The responses for the attitude questions were rated as: (1) Agree, (2) uncertain, and (3) Disagree. The correct answers were scored as 1 (Supplementary File S2).

#### Knowledge on malnutrition

The questionnaire on knowledge about malnutrition consists of 10 items, which include causes, clinical features, feeding practices, dietary requirements, and preventive measures. The level of knowledge was calculated using the scoring system; the correct answer was scored 1, and the wrong one was scored 0, the total score was then calculated (Supplementary File S3).

### Assessment of child oral health and nutritional Status

All clinical examinations were conducted by trained and calibrated personnel. Oral health assessments were performed by trained dentists using mouth mirrors and CPI probes, following standard clinical examination procedures. Dental caries experience was recorded using the deft index for primary dentition and the DMFT index for permanent dentition, as appropriate for the child's age. Caries severity was further assessed using the International Caries Detection and Assessment System (ICDAS). Oral hygiene status was evaluated using the Oral Hygiene Index–Simplified (OHI-S)**.**

Nutritional status was assessed by trained health personnel using standardized anthropometric techniques. Standing height, weight, mid upper arm circumference and head circumference were measured using stadiometers, digital weighing machines, and non-stretchable measuring tapes, respectively.

### Intervention Components

The intervention was delivered through a Common Risk Factor Approach (CRFA) and included the following components:

- Nutritional Counselling: Delivered by trained nutritionists, this included one-on-one counselling, group sessions using focus group discussions, and the distribution of IEC (Information, Education, and Communication) materials such as pamphlets and flipcharts tailored to local needs.
- Oral Health Education and Care: Provided by trained dental professionals, including oral hygiene education, supervised brushing, and distribution of oral hygiene kits (toothpaste and toothbrush). Curative and preventive dental treatments were administered free of cost, including restorations, extractions, fluoride varnish application, pit and fissure sealants, and caries-arrest measures such as silver diamine fluoride application.

Follow-up was conducted at a 6-month interval post-intervention to assess changes in outcomes.

### Ethics statement

The study was approved by the Institutional Ethics Committee (ECASM-AIMS-2023-154 dated 11th April 2023). Written informed consent was obtained from the caregivers while verbal assent was obtained from the children included in the study.

### Statistical analysis

Descriptive statistics were reported as mean ± SD for continuous variables and frequency for categorical variables. Reduction of dental caries experience (deft) in preschool and school age children at the 6-month intervals (pre and post intervention) was assessed by paired t- test.

Anthropometric assessment was carried out using World Health Organisation (WHO) growth standards, with age-specific software. For children aged less than 5 years (0–59 months), WHO Anthro software was used to compute weight-for-age (WAZ), length/height-for-age (HAZ), weight-for-length/height (WHZ), and BMI-for-age (BAZ) Z-scores based on WHO Child Growth Standards. For children aged 5 years and above, WHO AnthroPlus software was used to calculate weight-for-age, height-for-age, and BMI-for-age Z-scores. Children were classified as stunted, underweight, or wasted based on WHO cut-offs (Z-score < −2 SD).

Data were entered into Microsoft Excel and analysed using SPSS software (version 23.0). *P*-value < 0.05 is considered to be statistically significant.

## Results

### Demographic and baseline data

A total of 95 children (49 girls and 46 boys) were included in the study. Table 1 outlines the sociodemographic details of the study participants and caregivers enrolled in the study. The mean age of the children was 4.47 ± 1.42 years, while the mean age of the caregivers was 29.18 ± 5.05 years. Educational status varied among caregivers, with 36.3% educated up to middle school, while 24.2% and 23.1% were educated up to primary and high school, respectively. The majority of caregivers (93.1%) reported sources of income other than salaried employment. In terms of family details, the mean monthly income was INR 5,000 (IQR 4,000–8,000), with majority (69.1%) having more than one child. Regarding prenatal and natal history, the mean maternal age at the birth of the child was 23.6 ± 4.0 years, the mean birth weight was 2.4 ± 0.4 kg, and history of low birth weight was reported in 34.7% children. Notably, majority (58%) of the enrolled children were malnourished.

**Table 1:** Demographic and baseline data.

Domain	Variable	Characteristics	*n*	%
Demographic details	Age of child (in years)	Mean Age	4.47 ± 1.42	–
	Gender of child	Boy	46	48.4%
		Girl	49	51.6%
	Age of caregiver (in years)	Mean Age	29.18 ± 5.05	–
	Educational status of caregiver	Illiterate	6	6.6%
		Primary school	22	24.2%
		Middle school	33	36.3%
		High school	21	23.1%
		Diploma	5	5.5%
		Graduate	4	4.4%
	Source of income of caregiver	Other	81	93.1%
		Salaried employment	6	6.9%
	Relationship of caregiver to child	Mother	82	90.1%
		Father	6	6.6%
		Others	3	3.3%
Family details	Monthly income (In INR)	Median income	5,000 (IQR 4,000–8,000)	–
	Total number of children	One	29	30.9%
		More than one	65	69.1%
	Number of under-5 children in the family	One	47	71.2%
		More than one	19	28.7%
Pre-natal and natal history	Maternal age at birth of child (in years)	Mean age	23.6 ± 4.0	–
	Prenatal - Medical history	No	70	79.5%
		Yes	18	20.5%
	Prenatal - Drug history	No	76	86.4%
		Yes	12	13.6%
	Natal history - Delivery	Full term	71	78.9%
		Premature	19	21.1%
	Natal history – Type of delivery	Caesarean	30	33.3%
		Normal	60	66.7%
	Natal history - Place of delivery	Home	10	11.2%
		Hospital	79	88.8%
Child history	Birth weight (in kg)	Mean birth weight	2.4 ± 0.4	–
	Low birth weight	Yes	33	34.7%
		No	62	65.3%
	Malnourished	Yes	55	57.8%
		No	40	42.2%
	Natal history-Any condition of child at birth	Yes	10	11.1%
		No	80	88.9%
	Was child frequently having diseases during infancy	Yes	7	8.1%
		No	79	91.9%
	Past medical history	Yes	13	13.6%
		No	82	86.4%

### Knowledge, attitude and practices related to childhood dental caries, nutritional status and oral health

#### Childhood dental caries

The knowledge score significantly increased from 3.14 (pre-intervention) to 11.67 (post-intervention). The attitude score also showed a significant improvement, rising from 2.20 to 5.73. The practice score also increased significantly from 2.09 to 4.56. The overall KAP score increased significantly from 7.43 to 21.95 after the intervention (*p* < 0.001; Table 2).

**Table 2:** Mean KAP scores before and after intervention.

Questionnaire	Domain	Time point	*N*	Mean ± SD	MD ± SD	*p* value
KAP regarding childhood dental caries	Knowledge	Baseline	95	3.14 ± 2.29	8.53 ± 2.92	< 0.001*
		Post intervention	95	11.67 ± 1.51		
	Attitude	Baseline	95	2.20 ± 1.14	3.52 ± 1.63	< 0.001*
		Post intervention	95	5.73 ± 0.93		
	Practice	Baseline	95	2.09 ± 1.05	2.46 ± 1.24	< 0.001*
		Post intervention	95	4.56 ± 0.82		
	KAP score	Baseline	95	7.43 ± 3.84	14.52 ± 5.01	< 0.001*
		Post intervention	95	21.95 ± 2.80		
Knowledge regarding nutrition	Knowledge	Baseline	95	6.22 ± 3.43	3.67 ± 3.42	< 0.001*
		Post intervention	95	9.89 ± 1.02		
KAP regarding Child Oral Health	Knowledge	Baseline	95	3.32 ± 2.56	6.57 ± 2.71	< 0.001*
		Post intervention	95	8.94 ± 1.45		
	Attitude	Baseline	95	3.05 ± 1.43	1.71 ± 1.53	< 0.001*
		Post intervention	95	4.77 ± 0.79		
	Practice	Baseline	95	3.61 ± 1.51	1.64 ± 1.74	< 0.001*
		Post intervention	95	5.25 ± 0.88		
	KAP score	Baseline	95	9.97 ± 4.85	8.87 ± 5.24	< 0.001*
		Post intervention	95	18.95 ± 2.53		

KAP, knowledge, attitude and practice. *p-Value significant at ≤ 0.05.

#### Nutritional status

The caregivers' knowledge regarding nutrition improved significantly, with the score increasing from 6.22 to 9.89 after the intervention (Table 2).

#### Child oral health

The knowledge score regarding child oral health showed a remarkable increase from 3.32 to 8.94 post-intervention while the caregivers' attitudes towards child oral health also improved significantly, with the score rising from 3.05 to 4.77, post intervention. The practice score related to child oral health increased significantly from 3.61 to 5.25 after the intervention. The overall KAP score increased significantly from 9.97 to 18.95 after the intervention (*p* < 0.001; Table 2).

### Caries and oral hygiene status before and after intervention

Significant improvement was noted in the dental caries status (as recorded by dt, ICDAS_0, and ICDAS_1 indices) post-intervention, compared to baseline (*p* = 0.002, *p* = 0.031 and *p* < 0.001, respectively; Table 3). The DMFT index was not included in the analysis, as only a small proportion of participants were older than six years.

**Table 3:** Comparison of oral health status before and after intervention.

Oral health Condition	Index	Time point	*N*	Mean	Std. Deviation	*p* value
Dental caries status	dt	Baseline	7	7.71	3.59	0.002*
		Post intervention	7	1.43	0.53	
	ft	Baseline	0	.	.	–
		Post intervention	46	4.54	2.92	
	et	Baseline	1	2.00	.	–
		Post intervention	1	2.00	.	
	deft	Baseline	77	2.69	3.07	0.339
		Post intervention	77	2.82	3.39	
	ICDAS 0	Baseline	7	11.00	3.958	0.031*
		Post intervention	7	17.14	3.024	
	ICDAS 1	Baseline	19	6.89	2.514	< 0.001*
		Post intervention	19	4.26	2.705	
	ICDAS 2	Baseline	.	0	.	–
		Post intervention	.	0	.	
	ICDAS 3 and 4	Baseline	2	2.50	.707	0.205
		Post intervention	2	1.00	.000	
	ICDAS 5 and 6	Baseline	2	8.00	5.657	0.314
		Post intervention	2	1.50	.707	
Oral hygiene status	OHIS	Baseline	84	0.86	0.87	0.05
		Post intervention	84	0.57	0.49	

dt, decayed teeth; et, extracted teeth due to caries; ft, filled teeth; ICDAS, International Caries Detection and Assessment System; OHIS, Oral Hygiene Index Scores. *N* = Number of children with the condition. *p-Value significant at ≤ 0.05.

### Anthropometric outcomes

Among children aged less than 5 years, the weight-for-age Z-score improved significantly from a median of −1.70 (IQR: −2.47, −0.87) at baseline to −1.54 (IQR: −2.24, −0.77) post-intervention (*p* = 0.024). The weight-for-length Z-score showed a marked increase from −1.97 ± 1.28 to −1.24 ± 1.11 (*p* < 0.001) (Table 4).

**Table 4:** Pre- and post-intervention comparison of anthropometric Z-scores (*n* = 50) for children aged less than 5 years.

Variable	Pre-intervention	Post-intervention	Test statistic	*p*-value
ZLEN (Length/Height-for-age)	−1.04 (−1.73, 0.52)	−1.27 (−2.02, 0.04)	W = 1,260	<0.001*
ZWEI (Weight-for-age)	−1.70 (−2.47, −0.87)	−1.54 (−2.24, −0.77)	W = 403	0.024*
ZBMI (BMI-for-age)	−1.93 ± 1.34	−1.10 ± 1.15	t = −8.30	<0.001*
ZWFL (Weight-for-length)	−1.97 ± 1.28	−1.24 ± 1.11	t = −7.68	<0.001*

*p-Value significant at ≤ 0.05.

Among children aged 5 years and above, weight-for-age Z-scores showed a small, non-significant improvement from −1.65 to −1.50 (*p* = 0.09), while height-for-age Z-scores declined significantly from −0.72 to −1.32 (*p* < 0.001) (Table 5).

**Table 5:** Comparison of Pre- and post-intervention anthropometric Z-scores (*n* = 45) for children aged 5 years and above.

Variable	Pre-intervention Median (IQR)	Post-intervention Median (IQR)	Wilcoxon W	*p*-value
WAZ (Weight-for-age Z-score)	−1.65 (−2.42, −0.78)	−1.50 (−2.31, −0.83)	367	0.09
HAZ (Height-for-age Z-score)	−0.72 (−2.19, 0.01)	−1.32 (−2.47, −0.56)	1,029	<0.001*
BAZ (BMI-for-age Z-score)	−1.57 (−2.94, −0.97)	−1.06 (−1.75, −0.23)	126	<0.001*

*p-Value significant at ≤ 0.05.

## Discussion

The Common Risk Factor Approach (CRFA) implemented in the present study demonstrated significant effects across interconnected domains of childhood dental caries, nutrition, and overall growth among indigenous children in Kerala, underscoring its relevance as a population-level prevention strategy. The observed improvements in caregivers' knowledge, attitudes, and practices (KAP) related to oral health, nutrition, and child care reflect successful engagement with key proximal implementers and highlight the feasibility of embedding CRFA-based interventions within community and primary healthcare platforms. Enhanced caregiver awareness, combined with the delivery of preventive and curative dental services, translated into measurable improvements in dental caries outcomes, supporting the effectiveness of integrated, multicomponent delivery models in resource-constrained settings.

From an implementation perspective, these findings reinforce the utility of CRFA in addressing shared behavioural and social determinants of oral health and nutrition. The established bidirectional relationship between dental caries, nutritional status, and child growth suggests that siloed interventions are unlikely to achieve sustained impact. Evidence indicates that dental caries may adversely affect dietary intake and growth through pain, infection, and feeding difficulties (28).

Literature evidence further demonstrates a positive association between dental caries in the primary dentition and both wasting and stunting (29). Conversely, children with borderline undernutrition (adjusted OR 2.05; 95% CI: 1.20–3.49) and undernutrition (adjusted OR 3.46; 95% CI: 1.93–6.29) face a substantially higher risk of dental caries, with progressively higher mean deft scores across worsening nutritional categories (28) These findings underscore the need for integrated prevention strategies that simultaneously address oral health and nutrition within routine child health services.

Evidence from systematic reviews and meta-analyses indicates that oral health promotion programmes are not only clinically effective in reducing DMFT scores but are also cost-effective, contributing to reductions in downstream treatment costs and healthcare burden (30). In policy terms, the current findings support the incorporation of CRFA into existing maternal and child health, nutrition, and school health programmes, with clear pathways for intersectoral collaboration between dental services, nutrition programmes, and primary healthcare systems. Institutionalising CRFA through guidelines, workforce training, and performance indicators may facilitate scalability and sustainability, particularly in underserved and indigenous populations, and advance equitable, integrated primary healthcare delivery.

The Common Risk Factor Approach–based intervention resulted in meaningful improvements in anthropometric indicators, particularly among children under five years of age. Significant gains in weight-for-age (WAZ) and weight-for-length (WHZ) Z-scores indicate reductions in underweight and wasting, as per WHO cut-offs (Z-score < −2 SD). The observed improvements are consistent with findings from large community-based nutrition education interventions. A multicomponent intervention among tribal children in Maharashtra demonstrated substantial reductions in wasting and underweight following sustained caregiver counselling and convergence with primary health services (31). Similarly, a health and nutrition education programme in Gujarat, delivered over a 16-month period, reported significant declines in underweight (39% to 32%) and wasting (20% to 13.6%) (32).Comparable findings have also been reported from a 3-month community-based nutrition education intervention in Ghana, where significant improvements were observed in underweight and wasting, while linear growth remained unchanged (33).

In contrary, among children aged five years and above, improvements in weight-for-age were modest, while height-for-age (HAZ) Z-scores declined significantly, reflecting persistent stunting. Notably, even a 2-year community-based nutrition education programme in South Africa failed to demonstrate meaningful catch-up growth in height-for-age despite improvements in weight-related indicators (34). WHO defines stunting as a manifestation of chronic undernutrition resulting from prolonged nutritional deprivation and recurrent infections, particularly during the first 1,000 days of life (35). Height-for-age Z-scores represent cumulative linear growth relative to age-specific WHO standards and are strongly influenced by early-life nutritional and infectious exposures (36). The application of height-for-age–Z score based metrics to evaluate intervention effects are insufficient to infer group-level linear catch-up growth, as age-standardized HAZ trajectories may remain stable or decline despite absolute gains in stature when growth velocity does not meet reference expectations (37).

A recent systematic review has highlighted the close and bidirectional relationship between oral health and general health (38), demonstrating how oral conditions share common risk factors with major non-communicable diseases and can influence overall health outcomes. Despite this evidence, oral health remains one of the most neglected aspects of healthcare in many countries, particularly within primary care systems. Integrating oral health into primary healthcare can substantially enhance accessibility, equity, and affordability of oral care services. Approaches such as interprofessional collaborative practice, interprofessional education, public–private partnerships, and closed-loop referral mechanisms have been proposed as effective strategies to facilitate this integration.

The Common Risk Factor Approach (CRFA) provides an effective policy framework for integrating oral health with general health by addressing shared determinants such as diet and hygiene. Strengthening interprofessional collaboration particularly between dental professionals, nutritionists, and primary healthcare providers can facilitate the delivery of preventive dental and nutrition interventions at the community level. The observed improvements in caregivers' knowledge, attitudes, and practices, alongside reductions in dental caries and improvements in nutritional status, highlight the value of this integrated approach.

Policy efforts should focus on incorporating CRFA into dental education, continuing professional development, and primary healthcare programs to improve awareness and uptake among professionals. Embedding CRFA within existing public health and nutrition initiatives can enhance efficiency, sustainability, and equity in oral healthcare delivery.

### Strength and limitations of the study

One of the key strengths of this study is its integrated, community-based intervention that simultaneously addressed oral health and nutrition using a Common Risk Factor Approach. The study used standardised and validated assessment tools, including WHO growth standards and dental indices, at both baseline and follow-up.

This study has several limitations that should be acknowledged. The absence of a control or matched comparison group limits the ability to attribute the observed improvements solely to the intervention, as changes could also reflect maturation effects, external influences, or social desirability bias. The assessment of knowledge, attitude, and practices (KAP) was based on self-reported responses from caregivers, which may introduce measurement bias due to recall errors or the tendency to provide socially desirable answers, particularly following the intervention. Additionally, as the study was conducted at a single health center in Kerala with a relatively small sample size, the findings may not be generalizable to other regions, indigenous communities, or socio-economic contexts.

## Conclusion

The outcomes of this study establish the effectiveness common risk factor approach involving an integrated medical-dental model in improving childhood oral health and malnutrition. Approaches such as this should be implemented at community level to improve dental health among children which would help improve the general health and hence reduce the healthcare burden while improving the productivity.

## Data Availability

The raw data supporting the conclusions of this article will be made available by the authors, without undue reservation.

## Supplementary material

Supplementary File S1 Questionnaire- KAP on KAP dental caries.

Supplementary File S2 Questionnaire-KAP on child oral health.

Supplementary File S3 Questionnaire-Knowledge on nutrition.
