# Supplementary material for: A common risk factor strategy for combating childhood oral diseases and malnutrition in Kalpetta, India
Source: Front Oral Health. 2026 Feb 16;7:1673066. doi: 10.3389/froh.2026.1673066 (PMC12950718; doi:10.3389/froh.2026.1673066)
Supplement: Supplementary File S1 — Questionnaire- KAP on KAP dental caries. [file Table1.docx]

Common Risk Factor Approach for improving early childhood oral health and malnutrition among primary and pre-school children in Kalpetta, India

**Knowledge, Attitude and Practice among mothers to prevent Early childhood caries**

*Study ID:*

| **Knowledge items** | | **Yes** | **No** | **Don’t know** |
| --- | --- | --- | --- | --- |
| 1. | Quantity of toothpaste in children < 3 years |  |  |  |
| 2. | Quantity of toothpaste in children > 3 years |  |  |  |
| 3. | Mothers’ diet during pregnancy affects development of baby’s teeth |  |  |  |
| 4. | Canned juice can be given frequently to your child |  |  |  |
| 5. | Dental checkup at first year of your child life is important even if child does not have tooth decay or tooth pain |  |  |  |
| 6. | Feeding your child with baby bottle at nighttime has an influence on child teeth |  |  |  |
| 7. | Fluoridated toothpaste helps in preventing your child tooth decay |  |  |  |
| 8. | Controlling sugary intake frequency during the day can affect tooth decay |  |  |  |
| 9. | Appearance of white lines or white spots on the surfaces of the teeth are the first signs of tooth decay |  |  |  |
| 10. | Germs that cause tooth decay can be transmitted from mother to her child by kissing on his lips/chewing food by mother before giving it to her child |  |  |  |
| 11. | Decay in baby teeth can harm the future of new adult teeth |  |  |  |
| 12 | the care of the oral health and oral hygiene is important for the health of the permanent teeth |  |  |  |
| 13. | Tooth decay in children is inherited |  |  |  |

| Attitude items | | Strongly disagree | Disagree | Neither agree nor disagree | Agree | Strongly disagree |
| --- | --- | --- | --- | --- | --- | --- |
| 1. | Maintaining the child oral health is the parent’s responsibility |  |  |  |  |  |
| 2. | Prolonged and frequent breast-feeding harm your child’s teeth |  |  |  |  |  |
| 3. | Baby teeth should be cleaned as soon as it erupts |  |  |  |  |  |
| 4. | Providing fresh juices frequently during the day can harm your child’s teeth |  |  |  |  |  |
| 5. | Taking the child for dental check-up as soon as his/her teeth erupt |  |  |  |  |  |
| 6. | Brushing the children’s teeth at the age of 6 years and below should be done under the help of parents/caregiver |  |  |  |  |  |

| Practice items | | Strongly disagree | Disagree | Neither agree nor disagree | Agree | Strongly disagree |
| --- | --- | --- | --- | --- | --- | --- |
| 1. | Balanced diet is necessary for your child oral health |  |  |  |  |  |
| 2. | Providing breast feeding /bottle feeding during bed-time could harm your child teeth |  |  |  |  |  |
| 3. | Tooth decay can be transmitted by sharing utensils (i.e., spoons, forks) |  |  |  |  |  |
| 4. | Parents should make an effort to improve their knowledge in oral health |  |  |  |  |  |
| 5. | Cleaning your child’s teeth after each meal is necessary |  |  |  |  |  |

*(Reference: Al-Jaber et al. Knowledge, Attitudes, and Practices of Parents on Early Childhood Caries in Qatar—A Questionnaire Study. Eur J Dent. 2022:16(3);669-679)*
