# Supplementary material for: A common risk factor strategy for combating childhood oral diseases and malnutrition in Kalpetta, India
Source: Front Oral Health. 2026 Feb 16;7:1673066. doi: 10.3389/froh.2026.1673066 (PMC12950718; doi:10.3389/froh.2026.1673066)
Supplement: Supplementary File S2 — Questionnaire-KAP on child oral health. [file Table2.docx]

Common Risk Factor Approach for improving early childhood oral health and malnutrition among primary and pre-school children in Kalpetta, India

**Knowledge, Attitude and Practices of mothers toward their children’s oral health**

*Study ID:*

| **Knowledge** | |
| --- | --- |
| 1. | How many milk teeth are there in a child’s mouth   - 10 - 12 - 20 - 28 - Don’t know |
| 2. | Does the tooth paste contain fluoride?   - Yes - No - I don’t know |
| 3 | What is the role of the fluoride in the tooth paste?   - Prevents tooth decay - Prevents gum problems - Gives freshness - I don’t know |
| 4. | What is the most common dental disease in the child?   - Tooth decay - Bleeding gums - Discolored tooth - I don’t know |
| 5. | Which of the following food items can lead to tooth decay?  Chocolates   - Bakery products - Soft drinks - All of the above - I don’t know |
| 6. | Which of the following do you think prevents the tooth decay?   - Restricting sweets - Tooth brushing - Regular dental visits - Fluoridated tooth paste - All the above - I don’t know |
| 7. | Causes for gum disease?   - Improper brushing - Tartar - All of the above - I don’t know |
| 8. | Which of the following do you think prevents the gum disease?   - Regular brushing and mouth wash - Professional cleaning - All of the above - I don’t know |
| 9. | Which of the following can lead to irregular teeth?   - Thumb sucking/tongue - Runs in the family - All of the above - I don’t know |
| 10. | Can irregularly placed teeth be aligned in the correct position?   - Yes - No - I don’t know |

| **Attitude** | |
| --- | --- |
| 1. | It is necessary to take the child for regular dental visits   - Agree - Uncertain - Disagree |
| 2. | Cleaning of the child’s teeth should be done by mothers   - Agree - Uncertain - Disagree |
| 3. | It is necessary to clean the child’s teeth after every meal   - Agree - Uncertain - Disagree |
| 4. | Milk teeth do not require good care as it is going to fall anyway   - Agree - Uncertain - Disagree |
| 5. | Good oral health is related to the good general health   - Agree - Uncertain - Disagree |
| 6. | Healthy milk teeth are essential for children to chew the food properly   - Agree - Uncertain - Disagree |

| **Practice** | |
| --- | --- |
| 1. | When was the child’s first dental visit?   - 6 months after birth - After the eruption of first milk tooth - 1 year after birth - Not yet visited |
| 2. | When do you take your child to visit the dentist?   - Only during problems - Every 6 months - Every 1 year - Not particular |
| 3. | When did you commence the cleaning of your child’s teeth?   - Soon after first milk tooth eruption - After 4-6 milk teeth eruption - After all milk teeth eruption - After first birthday of the child   Don’t remember |
| 4. | Which of the following aids are used to clean your child’s teeth?   - Finger - Tooth brush - Twig - Any other |
| 5. | How many times do you brush your child’s teeth?   - Once in a day - Twice in a day - After every meal - Not particular |
| 6. | When do you change your child’s tooth brush?   - Once in 15 days - Once in a month - Every 2-3 months - Once the bristles fray out - Not particular |
| 7. | What material do you use to clean your child’s teeth?   - Tooth paste - Tooth powder - Any others |
| 8. | Does your child rinse the mouth after eating/drinking?   - Yes - No - Sometimes - I don’t know |
| 9. | At what time do you give the sugary food items to your child?   - With meals - In between meals - Before going to bed - Not particular |

# *Reference article: Jian at al., 2014 Knowledge, attitude and practices of mothers toward their children′s oral health: A questionnaire survey among subpopulation in Mumbai (India)*
