# Supplementary material for: A common risk factor strategy for combating childhood oral diseases and malnutrition in Kalpetta, India
Source: Front Oral Health. 2026 Feb 16;7:1673066. doi: 10.3389/froh.2026.1673066 (PMC12950718; doi:10.3389/froh.2026.1673066)
Supplement: Supplementary File S3 — Questionnaire-Knowledge on nutrition. [file Table3.docx]

Common Risk Factor Approach for improving early childhood oral health and malnutrition among primary and pre-school children in Kalpetta, India

**To assess Knowledge among mothers to prevent malnutrition**

*Study ID:*

| **Knowledge** | | **Yes** | **No** | **Don’t Know** |
| --- | --- | --- | --- | --- |
| **1.** | Imbalanced diet is the cause of malnutrition | 1 | 0 | 0 |
| **2.** | Less weight for age is a clinical feature of undernutrition | 1 | 0 | 0 |
| **3.** | Every 3 months, height and weight must be checked for children aged 3–5 years | 1 | 0 | 0 |
| **4.** | Cereals, pulses, milk, fish, and green leafy vegetables are essential for the growth of children | 1 | 0 | 0 |
| **5.** | Breastfeeding should be initiated within half an hour of delivery | 1 | 0 | 0 |
| **6.** | Supplementary feeding should be initiated at 6 months of age | 1 | 0 | 0 |
| **7.** | Child should be hospitalized to treat severe malnutrition | 1 | 0 | 0 |
| **8.** | Delayed physical growth and impaired cognitive development are the complications of malnutrition | 1 | 0 | 0 |
| **9.** | Adequate breastfeeding, nutritious food, and regular deworming will prevent malnutrition in children | 1 | 0 | 0 |
| **10.** | Immunization of children is the best way to protect the child against infectious diseases | 1 | 0 | 0 |

*(Reference: Anas Abdulrahman Aljohani, Mujahid Abdulrahman Aljohani. The knowledge of mothers about children malnutrition and associated factors. Int. J Med. In Devel Countries. 2020:4(1);7-11)*
